# Oxidative stress disruption of receptor-mediated calcium signaling mechanisms

**DOI:** 10.1186/1423-0127-20-48

**Published:** 2013-07-12

**Authors:** Tso-Hao Tang, Chiung-Tan Chang, Hsiu-Jen Wang, Joshua D Erickson, Rhett A Reichard, Alexis G Martin, Erica K Shannon, Adam L Martin, Yue-Wern Huang, Robert S Aronstam

**Affiliations:** 1 Department of Life Science, National Taiwan Normal University, Taipei, Taiwan 116, Republic of China; 2 Department of Biological Sciences, Missouri University of Science & Technology, 400 W 11th St, Rolla MO 65409, USA

**Keywords:** Calcium signaling, Inositol trisphosphate (IP3), Muscarinic acetylcholine receptor, Oxidative stress, Phospholipase Cβ, Store-operated calcium entry (SOCE)

## Abstract

**Background:**

Oxidative stress increases the cytosolic content of calcium in the cytoplasm through a combination of effects on calcium pumps, exchangers, channels and binding proteins. In this study, oxidative stress was produced by exposure to *tert*-butyl hydroperoxide (tBHP); cell viability was assessed using a dye reduction assay; receptor binding was characterized using [^3^H]*N*-methylscopolamine ([^3^H]MS); and cytosolic and luminal endoplasmic reticulum (ER) calcium concentrations ([Ca^2+^]_i_ and [Ca^2+^]_L_, respectively) were measured by fluorescent imaging.

**Results:**

Activation of M3 muscarinic receptors induced a biphasic increase in [Ca^2+^]_i_: an initial, inositol trisphosphate (IP3)-mediated release of Ca^2+^ from endoplasmic reticulum (ER) stores followed by a sustained phase of Ca^2+^ entry (i.e., store-operated calcium entry; SOCE). Under non-cytotoxic conditions, tBHP increased resting [Ca^2+^]_i_; a 90 minute exposure to tBHP (0.5-10 mM ) increased [Ca^2+^]_i_ from 26 to up to 127 nM and decreased [Ca^2+^]_L_ by 55%. The initial response to 10 μM carbamylcholine was depressed by tBHP in the absence, but not the presence, of extracellular calcium. SOCE, however, was depressed in both the presence and absence of extracellular calcium. Acute exposure to tBHP did not block calcium influx through open SOCE channels. Activation of SOCE following thapsigargin-induced depletion of ER calcium was depressed by tBHP exposure. In calcium-free media, tBHP depressed both SOCE and the extent of thapsigargin-induced release of Ca^2+^ from the ER. M3 receptor binding parameters (ligand affinity, guanine nucleotide sensitivity, allosteric modulation) were not affected by exposure to tBHP.

**Conclusions:**

Oxidative stress induced by tBHP affected several aspects of M3 receptor signaling pathway in CHO cells, including resting [Ca^2+^]_i_, [Ca^2+^]_L_, IP3 receptor mediated release of calcium from the ER, and calcium entry through the SOCE. tBHP had little effect on M3 receptor binding or G protein coupling. Thus, oxidative stress affects multiple aspects of calcium homeostasis and calcium dependent signaling.

## Background

Reactive oxygen species, including superoxide and hydrogen peroxide, are continuously generated in cells as products of aerobic respiration, and play important roles in a plethora of physiological functions [1,2]. At high levels, however, reactive oxygen species compromise cell function and induce cell death. Calcium is one of the most potent, specific and tightly controlled cellular regulators, and virtually every calcium control mechanism in the cell is both sensitive to oxidative stress and able to modulate it [1,3-5]. Oxidative stress increases the cytosolic content of calcium in the cytoplasm through a combination of effects on calcium pumps, exchangers, channels and binding proteins [4]. For example, oxidative stress has been reported to affect plasma membrane Ca^2+^-ATPase, sarco/endoplasmic reticulum ATPase [4], the IP3 receptor [6,7], ryanodine receptors [8,9], store-operated calcium entry through Orai channels [5,10], and voltage-gated calcium channels [11]. Addition of H_2_O_2_ to cultured hippocampal cells releases Ca^2+^ from the endoplasmic reticulum and depresses phospholipase C-mediated signaling [12]. Many of these effects are biphasic, initial activation giving way to inhibition at higher concentrations, and often involve effects on the redox state of protein sulfhydryl groups.

We recently characterized the *in vitro* cytotoxicity of metal oxide nanoparticles [13-15]. This toxicity is associated with elevated oxidative stress (OS), oxidative DNA damage, and the up-regulation of certain genes involved in oxidative stress responses and apoptosis. Moreover, ZnO nanoparticle toxicity is mitigated by treatment with the antioxidant *N–*acetylcysteine. Exposure to nanoparticles also causes an increase in resting intracellular calcium levels in human bronchial epithelial cells [13]. These findings led us to examine the effects of ZnO nanoparticles on muscarinic signaling through the M3 receptor – phospholipase Cβ pathway [16] that involves IP3-stimulated calcium release from the ER and subsequent depletion-induced calcium entry (i.e., store-operated calcium entry; SOCE) [17-19]. ZnO nanoparticles produced a marked inhibition of SOCE and also elevated cytosolic calcium concentrations without compromising the homeostatic mechanisms that keep calcium concentrations at low levels [16]. The extent to which these actions reflected oxidative stress as opposed to specific chemical effects was not assessed, providing the impetus for the present study.

Accordingly, in the present study the influences of a cell permeant oxidant, *tert*-butyl hydroperoxide (tBHP), on M3-mediated signaling were determined at the levels of 1) receptor binding, activation, allosteric regulation and G protein coupling, 2) cytosolic and ER luminal calcium concentrations, 3) calcium release from the endoplasmic reticulum, and 4) ER depletion-stimulated calcium entry from the extracellular medium. This oxidative stress had minimal effects on proximal receptor events, increased cytosolic calcium, decreased ER calcium, depressed calcium release from the endoplasmic reticulum (under certain conditions), and was a potent and consistent inhibitor of SOCE.

## Methods

### Cell culture and cytotoxicity and ROS measurements

CHO cells expressing the human M3 muscarinic acetylcholine receptor open reading frame (cDNA Resource Center, http://www.cdna.org) were cultured in DME/F12 media containing 10% FBS and penicillin/streptomycin (100 IU/100 μg/ml) at 37° in a 5% CO_2_ humidified environment.

Cell viability was revealed by the ability of the cells to reduce MTS (3-(4,5-dimethylthiazol-2-yl)-5-(3-carboxymethoxyphenyl)-2-(4-sulfophenyl)-2H-tetrazolium, inner salt; Promega, Madison). Cells (≈ 7000) were seeded into wells of a 96-well plate and incubated for 16 h. The cells were exposed to tBHP for 30, 90 or 150 min, and then washed before determining fluorescence using a FLUOstar Omega plate reader (BMG Labtech).

The concentration of reactive oxygen species (ROS) was determined by the reduction of 2′,7′-dichlorodihydrofluorescein (DCFDA; Sigma). CHO-M3 cells were plated on a 96 well plate with opaque walls at a density of 30,000 cells/well, and incubated overnight in phenol red-free media. Following a 90 min exposure to tBHP, the media was removed and the cells were washed with BSS. DCFDA (100 μl of a 40 μM solution) was added, and the plate was incubated for 1 h before measuring fluorescence at 485 nm excitation/520 nm emission using a FLUOstar plate reader.

The level of peroxide in the medium was quantified by measuring Fe^2+^ reduction using the PeroxiDetect kit (Sigma) and a FLUOstar plate reader (absorbance at 560 nm).

### Muscarinic receptor binding

Muscarinic receptors were labeled using [^3^H]*N*-methylscopolamine ([^3^H]MS; PerkinElmer), a non-selective muscarinic antagonist. An aliquot (15–30 μg protein) of CHO-M3 cells homogenized in 50 mM Tris–HCl (pH 7.4), was incubated with [^3^H]MS in 50 mM Tris–HCl (pH 7.4) for 60 minutes at room temperature in a final volume of 1 ml. In some experiments, the cells were exposed to 2 mM tBHP for 90 min before harvesting. Binding was determined by filtering the suspension through glass fiber filters (1 μm pore size) and measuring the amount of radioactivity bound to membranes trapped on the filters by liquid scintillation counting. Non-specific binding was determined in the presence of 10 μM atropine.

[^3^H]MS binding parameters (receptor concentration, dissociation constant, dissociation rate constant) were determined by nonlinear regression (DeltaGraph) using a mass action expression for a single population of receptor binding sites. Binding parameters were averaged from 3 independent experiments. Agonist binding was determined by the ability of carbamylcholine to inhibit the binding of 0.3 nM [^3^H]MS. 5′-Guanylyl-imidodiphosphate (Gpp(NH)p) was included in some experiments to assess receptor – G protein interactions.

### Measurement of cytosolic calcium

Cytosolic calcium concentration ([Ca^2+^]_i_) was monitored using the calcium-sensitive fluorescent dye, fura-2 (Molecular Probes), in a ratiometric assay, as previously described [16]. Briefly, cells (120,000) were seeded onto 29 mm glass bottom dishes and incubated for 16 h. The cells were then incubated with 5 μM fura-2 AM for 60 min at room temperature in a basal salt solution (BSS; 130 mM NaCl, 5.4 mM KCl, 5.5 mM glucose, 2 mM CaCl_2_, 1 mM MgCl_2_, and 20 mM HEPES, pH 7.4). The cells were washed twice, and the incubation continued for an additional 30 min. When experiments were performed in the absence of extracellular calcium, the cells were switched to BSS lacking CaCl_2_ 3 minutes prior to beginning calcium measurements.

The tBHP exposure period followed the fura-2 loading period in the 30 min exposure paradigm, but encompassed the fura-2 loading period in both the 90 and 150 min exposure paradigms.

[Ca^2+^]_i._ was measured at ambient temperature using an InCyt Basic IM™ Fluorescence Imaging System (Intracellular Imaging, Inc., Cincinnati, OH) using an Olympus UPLanSApo Uis2 20x objective. The ratio of fluorescent signals at 512 nm after excitation at 340 and 380 nm reflected [Ca^2+^]_i._. The fluorescence from 18–25 cells was measured in each plate. Cells that did not maintain a constant resting [Ca^2+^]_i._ (1–2 cells/plate), or that completely failed to respond to carbamylcholine (< 1 cell/plate), were excluded from the analysis. Carbamylcholine (typically 10 μM) was added after a baseline level was recorded for 30–40 sec; the carbamylcholine remained in the medium throughout the measurement period (3–5 min).

### Measurement of endoplasmic reticulum calcium

The concentration of Ca^2+^ in the lumen of the ER ([Ca^2+^]_L_) was measured using the low-affinity calcium indicator, Mag-Fura 2 (Molecular Probes). Fluorescence at 512 nm was measured following excitation at 340 nm and 380 nm using the InCyt imaging system. CHO cells (160,000) were seeded onto 29 mm glass bottom plates and incubated at 37°C for approximately 18 hours. The media was removed and the cells rinsed with BSS and then incubated with Mag-Fura 2 (50 μM) for 60 minutes at room temperature. Mag-Fura 2 was removed and the cells incubated for an additional 30 minutes. Prior to imaging, the BSS was removed and replaced with Ca^2+^- and Mg^2+^-free BSS. During imaging, the cells were permeabilized by exposure to 0.005% saponin for 70 seconds to release cytosolic calcium. This caused a rapid decrease in total fluorescence intensity following excitation at both 340 and 380 nm, but elimination of the pervasive cytosolic signal allowed detection of ER lumen calcium levels 30 seconds after saponin addition. Following stabilization of the signal (≈100 sec), R_340/380_ was averaged for 10 seconds in approximately 20 cells on each plate.

### Statistical analysis

Parameters (calcium concentrations, dissociation constants; kinetic constants, receptor concentrations, IC50’s) from two populations were compared as the means from 3–20 independent experiments (with 3 replicates in each binding measurement protocol and 18–24 replicates in each calcium measurement) using Student’s t-test. Values from experiments with multiple independent variables were compared by ANOVA and Tukey’s test using GraphPad Prism software. Significant differences were indicated by P values of < 0.05.

## Results

The effects of oxidative stress on signal transduction in CHO cells expressing the human M3 muscarinic receptor were studied.

### Muscarinic receptor mediation of cellular calcium responses

**Figure 1:**
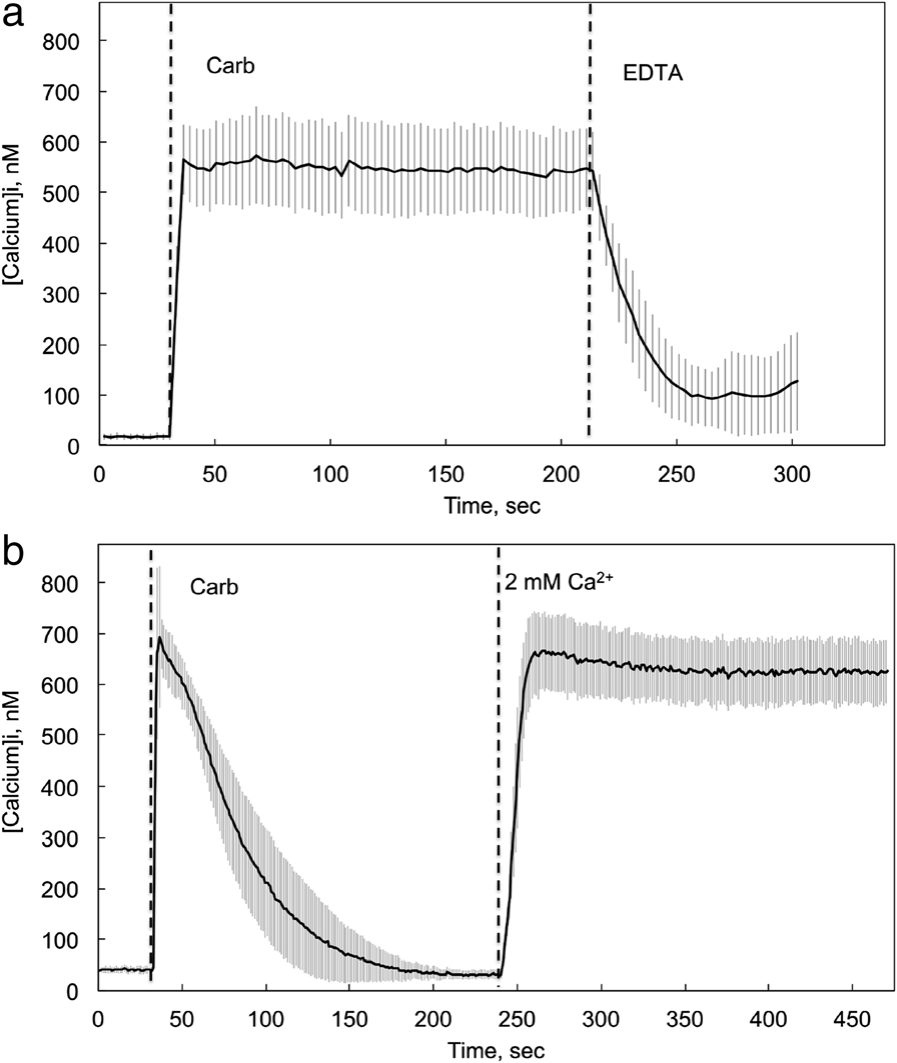
**The influence of cholinergic activation on cytosolic calcium levels in CHO-M3 cells.** CHO cells stably transfected with the gene for human M3 muscarinic acetylcholine receptor (CHO-M3) were loaded with fura-2 AM dye for 60 min at room temperature. The loading dye was removed and replaced with basal salt solution (BSS) and the cells were incubated for an additional 30 min. The BSS solution was replaced by fresh BSS or BSS without calcium immediately before measuring intracellular calcium levels. **a** Control responses measured in the presence of 2 mM extracellular calcium. Carbamylcholine (10 μM) was added at the time indicated by the first dashed vertical line. EDTA (5 mM) was added at the time indicated by the second dashed vertical line. **b** Control responses measured in the absence of extracellular calcium. Calcium was introduced into the extracellular medium (final concentration, 2 mM) at the point indicated by the second dashed vertical line. The average and standard deviation of the calcium concentrations from 22 (Figure 1a) or 20 (Figure 1b) cells from a single experiment are shown. These results are representative of responses from more than 20 independent experiments. In all of the experiments related in this report, the resting calcium level was 27 ± 8 nM (N = 19, including measurements from >350 cells). Two components of the calcium response to extracellular calcium are evident: a rapid initial response reflecting release from the endoplasmic reticulum and a plateau response dependent on the influx of extracellular calcium.

The resting cytosolic calcium concentration ([Ca^2+^]_i_) of CHO-M3 cells in BSS in this series of experiments was 25.6 ± 8.8 nM (mean ± S.D.; N = 18). Stimulation with the muscarinic agonist carbamylcholine (10 μM) led to a rapid and sustained increase in cytosolic calcium to a concentration that generally ranged from 500 to 700 nM (Figure 1a). The threshold concentration for elicitation of a response to carbamylcholine was approximately 0.3 μM. CHO cells not transfected with the M3 receptor gene did not respond to carbamylcholine. Moreover, the response of CHO-M3 cells to carbamylcholine was completely blocked by preincubation for 10 min with 10 μM atropine, a receptor antagonist. This demonstrates that the responses to carbamylcholine were mediated by the introduced M3 muscarinic receptor.

In the absence of extracellular calcium, the initial response to carbamylcholine remained intact, but [Ca^2+^]_i_ returned to baseline levels within a few minutes (Figure 1b). The initial response is generally thought to involve calcium release from the endoplasmic reticulum caused by activation of IP3 receptors [18,19]. The sustained increase in [Ca^2+^]_i_ depends on calcium entry from the extracellular medium [18,19]. Depletion of calcium from the ER is sensed by STIM1 proteins in the ER membrane, which triggers an interaction of STIM1 with Orai ion channels in the plasma membrane, and thus the entry of extracellular calcium (i.e., store-activated calcium entry; SOCE) [18,20]. This interpretation is supported by the present experiments insofar as chelation of extracellular calcium with EDTA eliminates the sustained increase in [Ca^2+^]_i_ (Figure 1a). Moreover, all responses to carbamylcholine were completely blocked by a 15 min preincubation with 50 μM 2-aminoethoxydiphenylborane, an IP3-receptor and SOCE channel inhibitor [18].

### Cytotoxicity of tBHP

**Figure 2:**
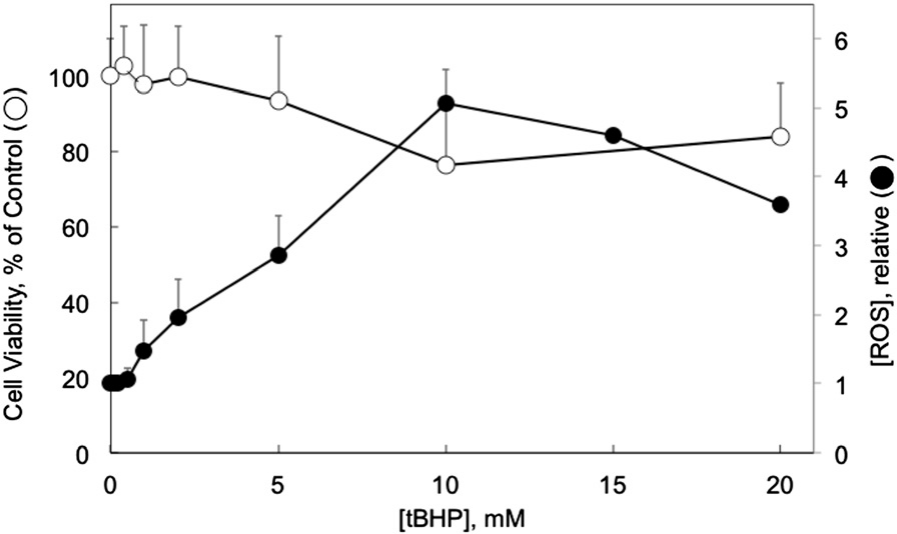
**tBHP induction of cytotoxicity and ROS.** CHO-M3 cells were exposed to tBHP at the concentrations indicated on the abscissa for 90 min. Cell viability (open circles) was determined using the MTS reduction assay. Relative concentration of ROS (control in the absence of tBHP = 1) was determined by the reduction of 2′,7′-dichlorodihydrofluorescein (CM-H_2_DCFDA) (closed circles). Points and bars indicate the means and standard deviations from 3 determinations.

CHO-M3 cell viability and the production of reactive oxidative species were revealed by the ability of the cells to reduce the fluorescent reagents MTS and CM-H_2_DCFDA, respectively. Exposure to tBHP induced cytotoxicity in a time and concentration-dependent manner. Following a 90 min exposure, cytotoxicity was not observed with tBHP concentrations up to 20 mM (Figure 2). However, when the exposure was extended for an additional hour, significant cytotoxicity was observed at concentrations as low as 1 mM (data not shown). Accordingly, subsequent experiments were performed using 90 min exposures to tBHP, i.e., conditions that produced high oxidative stress but did not eliminate the normal reducing capacity of the intracellular environment. Peroxide activity in the extracellular medium, as measured by oxidation of Fe^2+^ (PeroxiDetect; Sigma), did not decrease over the time courses of these experimental protocols.

The production of ROS in response to a 90 min exposure to tBHP is also illustrated in Figure 2. ROS was increased by up to 400% (at 10 mM tBHP) with a threshold tBHP concentration of 5 mM.

### Influence of tBHP on calcium responses to M3 muscarinic receptor activation

**Figure 3:**
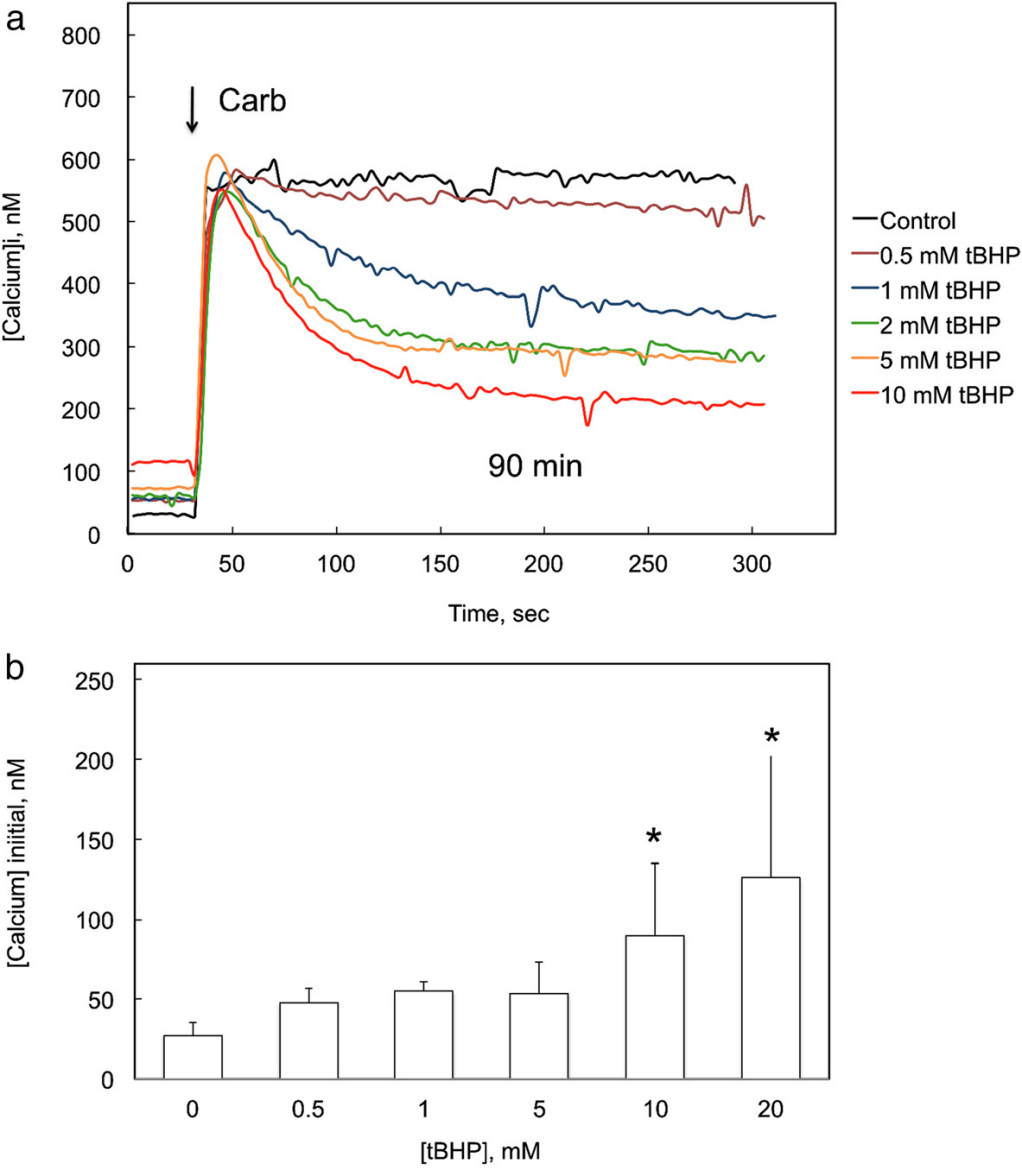
**Influence of exposure to tBHP on cholinergic receptor-mediated changes in cytosolic calcium. a** Intracellular calcium levels were measured in CHO-M3 cells following exposure to the indicated concentration of tBHP for 90 min, a period which included the fura-2 loading incubation, and tBHP was continually present during the calcium measurements. Carbamylcholine (50 μM) was added at the time indicated by the arrows. Two effects of tBHP on the calcium responses were apparent: an increase in resting calcium concentration to > 100 nM and a decrease in the plateau (SOCE-mediated) response. The results are the average from 19–25 cells from a representative experiment; each experiment was performed 3–6 times with essentially similar results. **b** Exposure to tBHP for 90 min increased resting cytosolic calcium concentration. Each column and bar represents the mean and standard deviation from 10 experiments, each including separate measurements from 15–25 cells. Calcium concentrations were measured in the presence of normal a (2 mM) concentration of extracellular calcium. Exposure to 10 and 20 mM tBHP increased [Ca^2+^]_i_ compared to control cells (*, p < 0.05; ANOVA and Tukey’s test).

M3 receptor-mediated responses to carbamylcholine were measured following exposure of CHO-M3 cells to tBHP for 90 min (Figure 3a). Following exposure to tBHP, the initial response to carbamylcholine was not significantly affected, indicating that muscarinic receptor-mediated release of calcium from the endoplasmic reticulum through IP3 receptors remained intact (Figure 3a). However, the ability of the cells to maintain the higher level of [Ca^2+^]_i_ was compromised in a concentration-dependent manner, suggesting a disruption of capacitive calcium entry from the extracellular medium (i.e., SOCE).

Resting intracellular calcium concentrations ([Ca^2+^]_i_) were increased following exposure to tBHP (Figure 3a). The increases in [Ca^2+^]_i_ following a 90 min exposure to various concentrations of tBHP measured in 10 independent experiments (15–25 cells/experiment) are summarized in Figure 3b. [Ca^2+^]_i_ was increased following exposure to 10 and 20 mM tBHP; following exposure to 20 mM tBHP, [Ca^2+^]_i_ was increased from 26 to 127 nM.

**Figure 4:**
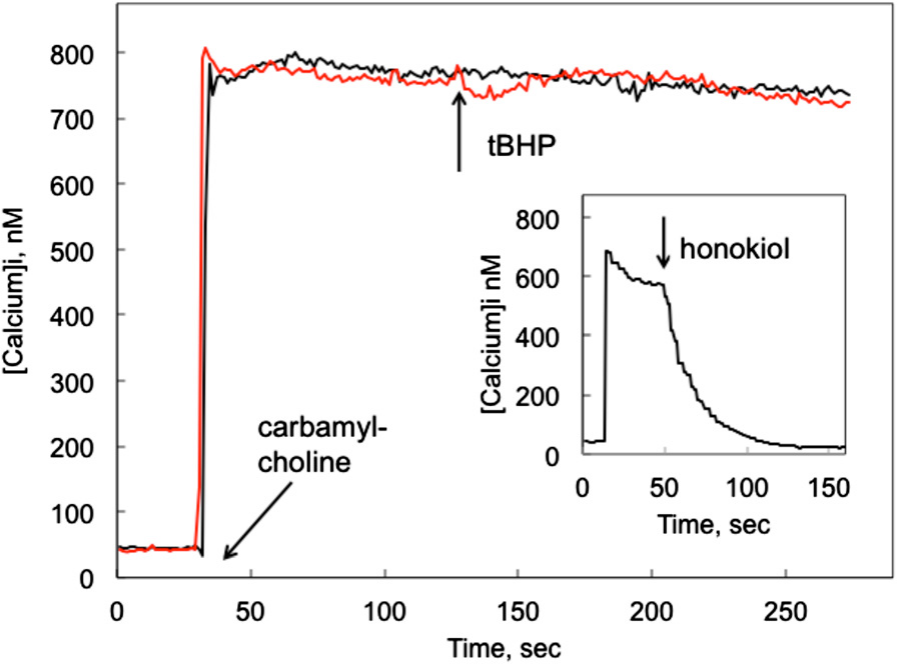
**Acute effect of tBHP on SOCE.** Fura-2-loaded CHO-M3 cells were stimulated with 10 μM carbamylcholine at the point indicated by the labeled arrow. tBHP (20 mM) was added at the 120 sec time point, as indicated by the arrow labeled “tBHP”. Under these assay conditions, the addition of direct inhibitors of SOCE channels, such as honokiol [21], 2-aminoethoxydiphenylborane, 1-(5-chloronaphthalenesulfonyl)homo piperazine hydrochloride (ML 9), and ZnO nanoparticles, cause an immediate reduction of SOCE; data from a typical experiment with 30 μM honokiol are presented in the insert for comparison purposes.

Acute exposure of CHO-M3 cells to tBHP following the activation of SOCE did not block calcium entry (Figure 4). In contrast, addition of certain direct SOCE channel inhibitors (e.g., 2-aminoethoxydiphenylborane, zinc oxide nanoparticles [16], and honokiol [21]; see insert to Figure 4) cause immediate reductions in SOCE (Aronstam et al., unpublished observations). This is consistent with the time-dependent effects of tBHP on production of ROS, and suggests that tBHP does not interact directly with the channel protein.

**Figure 5:**
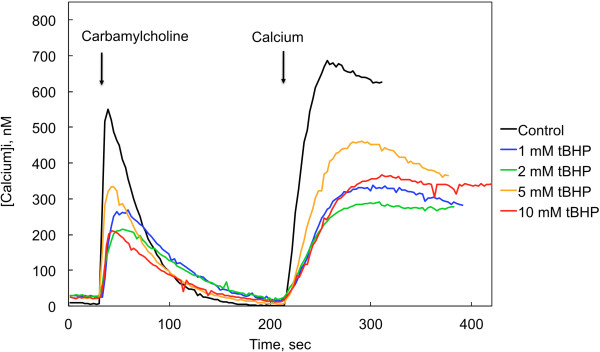
**Influence of tBHP on cholinergic receptor-mediated changes in cytosolic calcium measured in the absence of extracellular calcium.** Intracellular calcium levels were measured in CHO-M3 cells in the absence (Control) and presence of 1.0 – 10 mM tBHP (90 min), as indicated. Carbamylcholine (50 μM) was added at the time indicated by the first arrow. Calcium was reintroduced to the extracellular medium at the point indicated by the second arrow (final concentration = 2 mM). Results from a representative experiment involving 25 cells are shown; the experiment was performed 4 times with essentially similar results. Calcium responses (both initial peak response and plateau level response following calcium reintroduction of calcium) were significantly lower in the presence of any concentration of tBHP (1–10 mM); the corresponding responses following exposure to the different concentrations of tBHP (1 – 10 μM) were not different from each other (p > 0.05, ANOVA and Tukey’s test; N = 4).

M3 receptor-mediated responses to carbamylcholine were also measured in the absence of extracellular calcium (Figure 5). CHO-M3 cells responded with an initial increase in [Ca^2+^]_i_, representing release from the ER, that was not sustained. Reintroduction of calcium to the extracellular medium after the [Ca^2+^]_i_ had returned to a baseline level resulted in an immediate and sustained increase in [Ca^2+^]_i_ that reflected calcium entry via SOCE channels that had been activated by the carbamylcholine-induced depletion of calcium from the ER. The response to M3 receptor activation was depressed following a 90 min exposure to all tBHP concentrations at or above 1 mM (Figure 5). In contrast, initial IP3 receptor-mediated release observed in the presence of extracellular calcium was not depressed (Figure 3a). The initial rate of rise of [Ca^2+^]_i_ in response to carbamylcholine was 45 ± 8 nM/sec in control cells, and this rate was not significantly depressed after exposure to 1–10 mM tBHP (range: 23 – 41 nM/sec). However, the time course for the return to baseline [Ca^2+^]_i_ was consistently slower following exposure to tBHP (time constants for decay of [Ca^2+^]_i_ were increased from 17.2 ± 1.1 in control cells to 22–28 sec following exposure to tBHP; p < 0.05, controls compared to all tBHP treatment conditions, which were not different from each other), suggesting impairment of the calcium transporters that normally restore [Ca^2+^]_i_ to baseline levels [1]. tBHP caused a pronounced depression of calcium entry through the SOCE induced by the reintroduction of calcium to the extracellular medium (Figure 5). The initial rate of rise of [Ca^2+^]_i_ in response to reintroduction of calcium was 24 ± 8 nM/sec in control cells, significantly greater (p < 0.05; ANOVA and Tukey’s test) than the initial rates following treatment with any tBHP concentration from 1–10 mM (range: 5.6 – 10.5 nM/sec). However, the rates of calcium entry following reintroduction of calcium in cells exposed to the different concentrations of tBHP (1–10 mM) were not different.

### Influence of tBHP on thapsigargin-induced changes in cytosolic calcium concentrations

**Figure 6:**
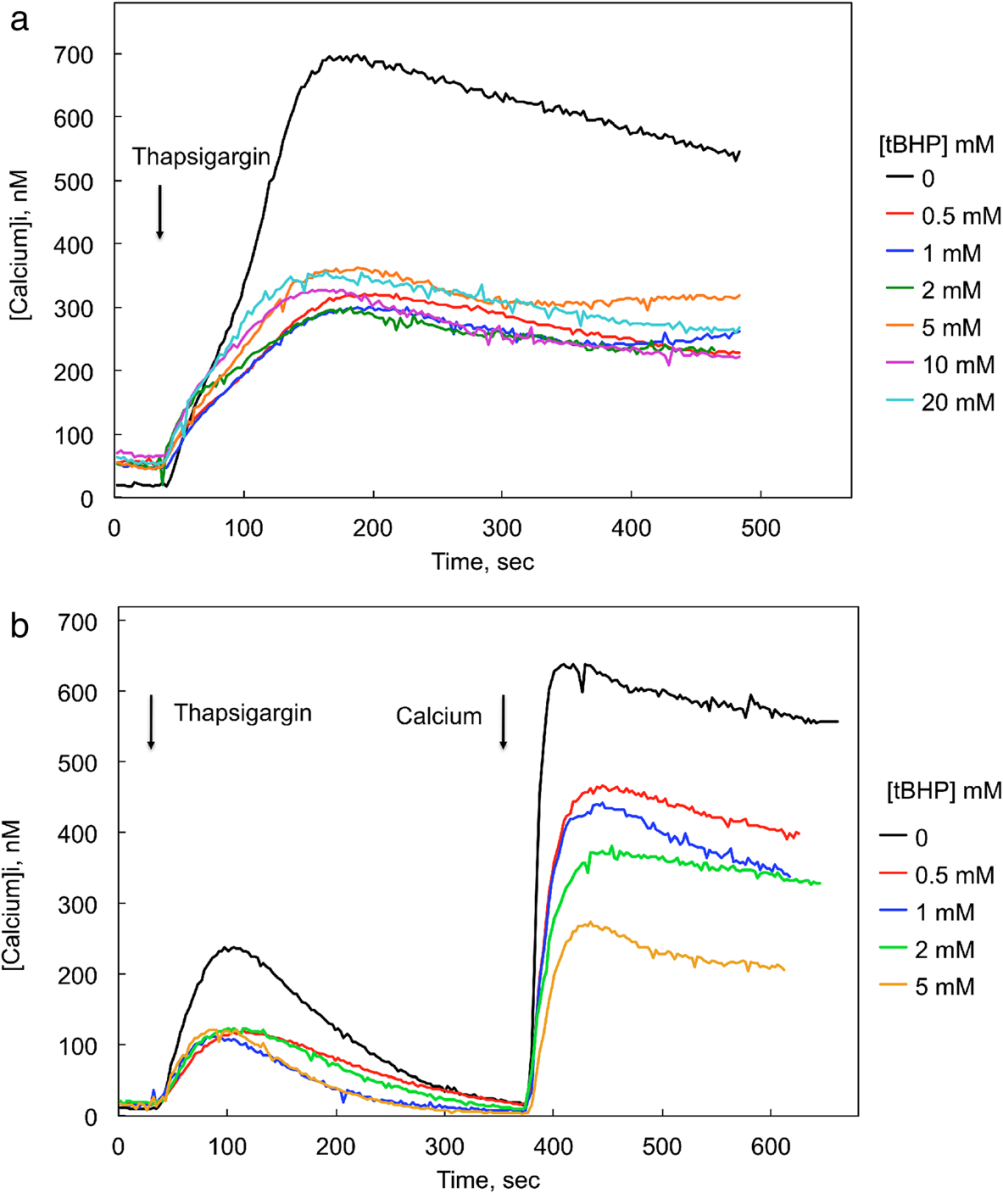
**Influence tBHP on thapsigargin-induced changes in cytosolic calcium concentrations in CHO-M3 cells measured in the presence and absence of extracellular calcium (2 mM).** Thapsigargin (1 μM) was added at the time indicated by the first arrow. Calcium concentration was determined following a 90 min exposure to the indicated concentrations of tBHP. Each line is the average response from 21–23 cells from two experiments; six replicate experiments yielded similar results. Thapsigargin-induced changes in cytosolic calcium concentrations were measured in the presence **(a)** and absence **(b)** of extracellular calcium. Calcium was reintroduced to the extracellular medium at the point indicated by the second arrow in **b** (final concentration = 2 mM). Cytosolic calcium levels measured 100 sec after exposure to thapsigargin were significantly lower in the presence of any concentration of tBHP (0.5- 20 mM), although the levels following exposure to the different concentrations of tBHP were not different from each other (ANOVA and Tukey’s test; p < 0.05, N = 6). Similarly, calcium levels 100 sec following reintroduction of calcium following thapsigargin treatment in absence of extracellular calcium **(b)** were significantly lower in the presence of any concentration of tBHP (0.5- 5 mM) (ANOVA and Tukey’s test; p < 0.05, N = 6), although the levels following exposure to the different concentrations of tBHP were not different from each other.

Thapsigargin (1 μM)-induced changes in [Ca^2+^]_i_ were measured in cells exposed to various concentrations of tBHP for 90 min (Figure 6). Thapsigargin inhibits the sarco/endoplasmic reticulum Ca^2+^-ATPases that transport calcium into the endoplasmic reticulum, thereby increasing cytosolic calcium levels. The consequent depletion of ER calcium activates SOCE, producing a sustained increase in [Ca^2+^]_i_. Exposure to tBHP for 90 min increased resting [Ca^2+^]_i_, although the initial response to thapsigargin was not clearly affected by tBHP (Figure 6a). The ability of this initial calcium release to evoke SOCE, however, was severely compromised (Figure 6a). The extent of this decrease was not concentration-dependent over the range examined (0.5 – 20 mM), suggesting a lower threshold concentration for this effect, or perhaps a limiting maximal effect. The initial rate of rise [Ca^2+^]_I_ in response to thapsigargin was 4.3 ± 0.6 nM/sec in control cells (i.e., one tenth the rate of rise in response to carbamylcholine), and this rate was not affected by exposure to 0.5 – 20 mM tBHP (range: 1.9 - 4.1 nM/sec).

IP3 receptor-mediated calcium release and SOCE were monitored by reintroducing extracellular calcium (2 mM) to CHO-M3 cells following exposure to thapsigargin (1 μM) in a calcium-free medium (Figure 6b). While thapsigargin depletes ER calcium, thereby opening the channels responsible for SOCE, calcium is not available to enter the cell until it is restored to the extracellular medium. In this paradigm, the initial thapsigargin-stimulated release of calcium from the ER was depressed following a 90 min exposure of CHO-M3 cells to tBHP; all tBHP concentrations from 0.5 to 5 mM caused a 50% inhibition of the initial response (Figure 6b). After [Ca^2+^]_i_ returned to baseline levels (about 6 min), calcium was added to the extracellular medium. SOCE visualized in this manner was depressed by 30-70% in a dose-dependent manner. The initial rate of rise [Ca^2+^]_I_ in response to reintroduction of calcium was 40.0 ± 5.1 nM/sec in control cells, more than twice the rates observed in cells exposed to 0.5 – 5 mM tBHP (range: 9 – 18 nM/sec; p < 0.05 for controls compared to cells compared to any concentration of tBHP; the rate differences among the different treatment groups were not significant).

### Influence of tBHP on endoplasmic reticulum calcium content

**Figure 7:**
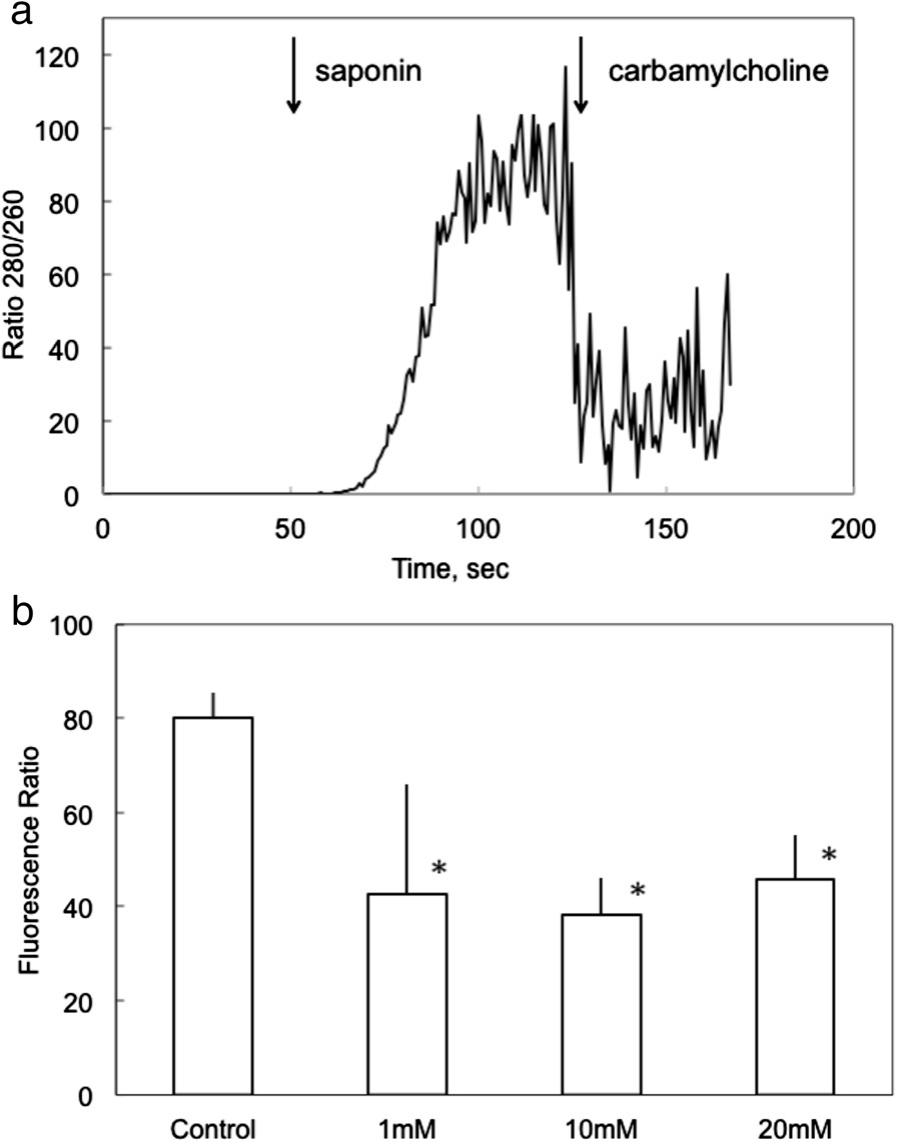
**Influence of tBHP on the concentration of calcium in the lumen of the ER.** The relative level of [Ca^2+^]_L_ was determined in cells in which cytosolic calcium had been depleted by permeabilization with 0.005% saponin. **a** Mag-Fura2 380/340 fluorescence ratio in a typical experiment. Saponin (0.005%) was added at the time indicated by the first arrow; carbamylcholine was added at the time indicated by the second arrow. Prior to permeabilization, the fluorescent signal associated with ER calcium is obscured by the dominant signal from cytosolic calcium. Data are the average from 20 cells in a typical experiment. [Ca^2+^]_L_ was inferred from the average ratio measured between 100 and 110 sec. **b** The ratio of fluorescence intensity of Mag-Fura2 following excitation of at 380 and 340 nm was determined in control cells and in cells exposed to 1, 10 or 20 mM tBHP for 90 min prior to imaging, as indicated. This ratio is an indication of [Ca^2+^]_L_. The column and bars represent the mean and standard deviation from 3 experiments that involved separate determinations in 20 cells. The asterisks indicate a statistical difference from the control level as determined by ANOVA with a Tukey post-test.

The influences of tBHP on [Ca^2+^]_i_ during the initial response to IP3 receptor- or thapsigargin-mediated release from the ER could reflect changes in the luminal concentration of calcium ([Ca^2+^]_L_). Accordingly, [Ca^2+^]_L_ was measured using an alternate calcium-sensitive dye (Mag-Fura2) and depletion of cytosolic [Ca^2+^]_I_ by permeabilization of the plasma membrane (Figure 7). The increase in ratio of fluorescent emission at 512 nm following excitation at 340 and 380 nm (R_340/380_) reflected ER Ca^2+^ levels insofar as: 1) R_340/380_ was immediately diminished following exposure to 10 μM carbamylcholine, and 2) pretreatment with the ER Ca^2+^-ATPase inhibitor, thapsigargin, prevented the increase in R_340/380_ observed upon permeabilization. Pretreatment with tBHP (1–20 mM) for 90 min caused a decrease in [Ca^2+^]_L_ to ≈ 55% of control levels.

### Influence of tBHP on muscarinic receptor ligand binding properties

**Figure 8:**
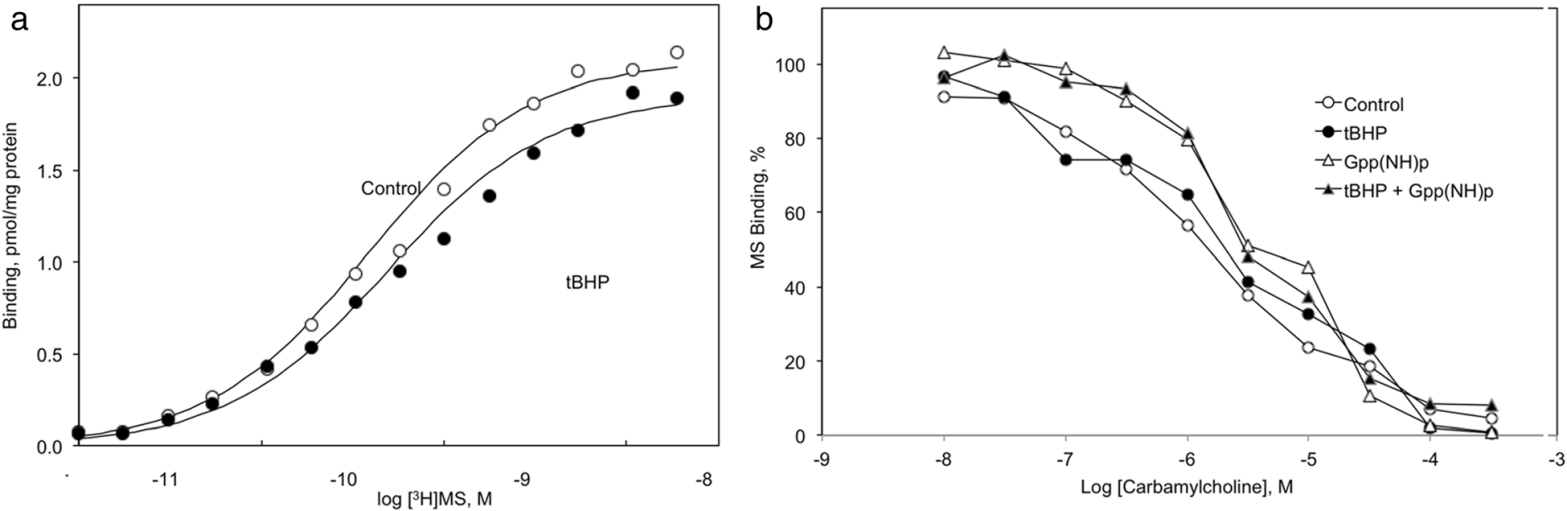
**Influence of tBHP on ligand binding to muscarinic receptors in CHO-M3 cells. a** The specific binding of [^3^H]*N*-methylscopolamine ([^3^H]MS) was measured at the concentrations indicated on the abscissa. The curve is drawn according to parameters derived from a nonlinear fit of the data to a model for ligand binding to a single population of independent receptors. Each data point reflects the mean from 3 experiments performed in triplicate. Binding parameters and experimental variation are presented in Table 1. Neither receptor concentration nor binding affinity was affected by exposure to tBHP (2 mM for 90 min). **b** The ability of carbamylcholine to inhibit the specific binding of 0.3 nM [^3^H]MS was determined in triplicate in control (open symbols) or tBHP-exposed (2 mM for 90 min) (closed symbols) M3-CHO cells in the absence (circles) or presence (triangles) of 10 μM Gpp(NH)p. Each point is the average of 3 measurements from 3 independent experiments. The ability of carbamylcholine to inhibit [^3^H]MS binding was not affected by a 90 min exposure to 2 mM tBHP (IC50’s = 1.8 ± 0.3 μM in control and 2.4 ± 0.6 μM in exposed cells). Carbamylcholine inhibition of [^3^H]MS binding was depressed in the presence of 5′-Gpp(NH)p (IC50’s = 5.0 ± 1.2 μM compared to 1.8 ± .03 mM in control cells). Exposure of the cells to tBHP did not affect guanine nucleotide depression of agonist binding affinity (IC50’s = 5.3 ± 0.5 μM and 2.4 ± 0.6 μM in exposed and control cells, respectively). Each point is the average of 3 measurements from 3 independent experiments.

The possibility that tBHP affects the muscarinic receptor component of the signal transduction pathway was evaluated using a specific receptor probe, [^3^H]MS. [^3^H]MS binding was well described by a binding isotherm for ligand binding to a single population of independent receptors (Figure 8a; Table 1). Receptor affinity for [^3^H]MS was high, nonlinear regression analysis revealed a dissociation constant of 0.39 ± 0.07 nM (Table 1), and binding affinity was not affected by exposure to 2 mM tBHP for 90 min. Receptor concentrations (2.1 ± 0.2 pmol/mg protein) were similarly not affected by exposure to tBHP.

**Table 1:** **Influence of tBHP on [**^**3**^**H]MS binding to human muscarinic receptors expressed in CHO cells**

**Receptor subtype**	**Treatment**	**K**_**D**_**, nM**	**[Receptor], pmol/mg protein**	**k**_**−1 **_**(min**^**-1**^**)**	**k**_**−1 **_**(min**^**-1**^**), with gallamine**
M3	Control	0.39 ± 0.07	2.1 ± 0.2	0.014 ± 0.002	0.009 ± 0.003
M3	tBHP	0.49 ± 0.10	1.9 ± 0.2	0.015 ± 0.003	0.010 ± 0.002
M2	Control			0.027 ± 0.004	0.004 ± 0.001
M2	tBHP			0.021 ± 0.006	0.005 ± 0.002

[^3^H]MS binding was measured in untreated cells (Control) or in cells exposed to 2 mM tBHP for 90 min before harvesting the cells for binding measurements.

K_D_, equilibrium dissociation constant; [Receptor], receptor density; k_−1_ rate constant for [^3^H]MS dissociation.

Mean ± S.D. (N = 3). None of the parameters measured after exposure to tBHP were significantly different from the corresponding control value. Gallamine decreased the dissociation rate constants (p < 0.05 with M3 receptors; p < 0.01 with M2 receptors); dissociation rate constants were not altered in cells exposed to 2 mM tBHP.

Agonist binding to muscarinic receptors reveals multiple aspects of receptor organization. Agonist binding was inferred from the ability of carbamylcholine to inhibit the binding of 0.3 nM [^3^H]MS to the receptor (Figure 8b). Carbamylcholine inhibition of [^3^H]MS was biphasic, displaying distinct high and low affinity components (approximately K_D_’s = 0.3 and 10 μM). The GTP analog, Gpp(NH)p, shifted the inhibition curves to the right, reflecting a conversion of receptors in a state characterized by a high affinity for agonists to a low agonist affinity conformation. These affinity states reflect the state of receptor coupling to transducer G proteins, with receptor-G protein complexes having a higher affinity for agonists than uncoupled receptors. The ability of carbamylcholine to inhibit [^3^H]MS binding was not affected by a 90 min exposure to 2 mM tBHP (IC50’s = 1.8 ± 0.3 μM in control and 2.4 ± 0.6 μM in exposed cells). Carbamylcholine inhibition of [^3^H]MS binding was depressed in the presence of 5’-Gpp(NH)p (IC50′s = 5.0 ± 1.2 μM compared to 1.8 ± .03 mM in control cells). Exposure of the cells to tBHP did not affect guanine nucleotide depression of agonist binding affinity (IC50’s = 5.3 ± 0.5 μM and 2.4 ± 0.6 μM in exposed and control cells, respectively). These results indicate that receptor-G protein uncoupling is not compromised by exposure to tBHP.

**Figure 9:**
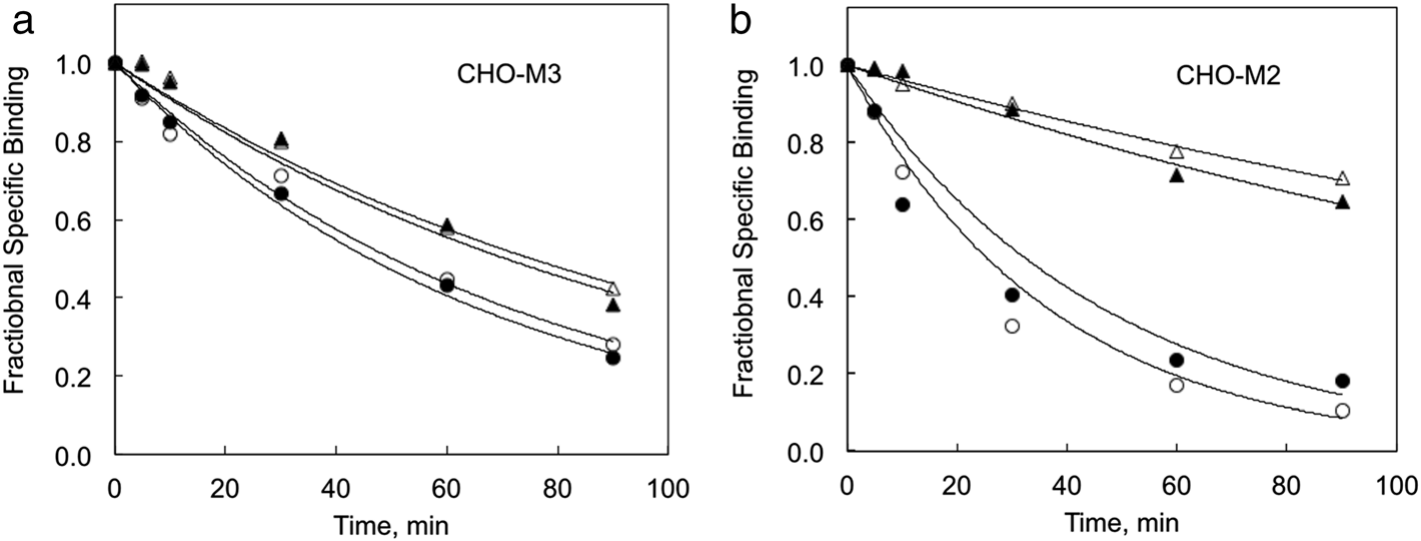
**The influence of tBHP on allosteric regulation of M3 muscarinic receptors expressed in CHO cells is illustrated. a** The dissociation of [^3^H]MS was measured by incubating CHO-M3 cell membranes with 1 nM [^3^H]MS for 1 hour before adding an excess of atropine (10 μM) to block the forward binding reaction. The off rate was measured in the absence (circles) and presence (triangles) of 10 μM gallamine in control (open symbols) or tBHP-exposed (2 mM 90 min) cells (closed symbols). Dissociation rate constants are listed in Table 1. **b** Similar responses in CHO cells expressing human M2 muscarinic receptors are shown. M2 receptors were examined because they typically display a more pronounced allosteric effect.

Muscarinic receptors can also be regulated by ligands that bind to an allosteric site. This allosteric effect is clearly evident in a decrease in the rate of [^3^H]MS dissociation in the presence of the allosteric ligand (e.g., [22]). Accordingly, we measured ligand dissociation from muscarinic receptors after exposure to 2 mM tBHP for 90 min (Figure 9). Exposure to tBHP had no effect on atropine-induced dissociation of an M3 receptor-[^3^H]MS complex in CHO-M3 cells, or on the ability of the allosteric ligand gallamine to slow the rate of dissociation (Figure 9a; Table 1). M2 muscarinic receptors display more pronounced allosteric effects than M3 receptors. Therefore, we also examined the influence of tBHP on allosteric muscarinic responses in CHO cells stably transfected with the human M2 muscarinic receptor (Figure 9b). Again, allosteric receptor actions were not affected by exposure to 2 mM tBHP for 90 min.

## Discussion

The influence of oxidative stress on muscarinic signaling pathways was investigated in CHO cells, which are widely used in the study of muscarinic signaling pathways. Wild type CHO cells do not express muscarinic receptors or acetylcholinesterase, but express the basic components of both the phospholipase Cβ [16] and adenylate cyclase [23] signaling pathways. CHO cells transfected with the different muscarinic receptor subtypes display expected ligand interactions and muscarinic pharmacology: Human M1, M3 and M5 receptors expressed in CHO cells stimulate the production of IP3, release calcium from the ER and activate SOCE; human M2 and M4 receptors expressed in CHO cells inhibit forskolin-stimulated cAMP formation.

Oxidative stress from endogenous or environmental stress affects multiple aspects of calcium metabolism [5,10]. For example, hydrogen peroxide was found to block SOCE calcium entry while stimulating active calcium transport in thyroid FRTL-5 cells in a phosphokinase C-dependent manner [24,25]. Similarly, tBHP increased resting [Ca^2+^]_i_. and selectively inhibited extracellular calcium entry in endothelial cells in response to bradykinin, while having a lesser effect on calcium release from the ER [26]. Bogeski et al. have provided electrophysiological evidence for H_2_O_2_ inhibition of Orai1, but not Orai3, activation [27]. The specific influences of redox state on calcium signaling depend on the cell type and time of exposure [5]. The present results are consistent with these earlier studies insofar as [Ca^2+^]_i_ and extracellular calcium entry (SOCE) were consistently depressed by oxidative stress. The present study extends these observations to the CHO cell model, and include concurrent measurements of oxidant effects on muscarinic receptor systems, immediate SOCE entry, ER calcium concentrations, and cytotoxic effects.

Activation of M3 receptor activates Gα_q_ proteins. Gα_q_, in turn, stimulates phospholipase Cβ which hydrolyzes the membrane phosphatidylinositol 4.5-bisphosphate releasing diacylglycerol and inositol trisphosphate (IP3). IP3 interacts with its cognate receptor on the endoplasmic reticulum to release stored calcium. This depletion of ER calcium induces an interaction between STIM proteins in the ER and Orai channels in the plasma membranes that allows calcium to enter the cell [17,18,28]. ER depletion-mediated calcium influx has been termed store-operated calcium entry (SOCE).

Four major effects of induction of acute oxidative stress were observed in the present study: 1) an increase in resting [Ca^2+^]_i._; 2) a decrease in [Ca^2+^]_L_; 3) a decrease in agonist-stimulated release of calcium from the ER mediated by IP3 receptors (in the absence of extracellular calcium); and 4) a pronounced decrease in SOCE (in the absence and presence of extracellular calcium). These effects are consistent with other accounts of oxidative stress increasing the cytosolic content of calcium [4] and depressing phospholipase C-mediated signaling [12].

Oxidative stress was induced by adding tBHP to the external medium. The insult was continuous; the level of peroxide in the medium did not change over the course of the experiments. Following exposure to 0.4-20 mM tBHP for 90 min, the cells remained attached to the glass and retained the ability to maintain a constant [Ca^2+^]_i._ and to reduce MTS. However, cytotoxicity was observed when the cells were exposed to these same concentrations of tBHP for longer periods of time (e.g., 150 min). In contrast, acute exposure to tBHP following the activation of SOCE did not block calcium entry (Figure 4), suggesting that tBHP actions are a consequence of developing oxidative stress and not a direct interaction with channel proteins.

The resting [Ca^2+^]_i._ in control cells was about 26 nM; exposure to 20 mM tBHP for 90 min increased this to 127 nM, although the cells remained capable of maintaining [Ca^2+^]_i_ at the new, higher [Ca^2+^]_i_ for at least one hour. This increase may involve impairment of transport processes or ion exchange mechanisms that determine calcium concentration [1].

The release of calcium from the ER was apparent in the initial release of calcium in response to carbamylcholine as well as in response to thapsigargin inhibition of ER calcium uptake. In the presence of extracellular calcium, this carbamylcholine-induced release was not affected by tBHP, even though the resting [Ca^2+^]_i._ was elevated (Figure 3). However, in the absence of calcium, the carbamylcholine-induced release of calcium from the ER was clearly reduced (Figure 5). Similarly, thapsigargin-induced net calcium release from the ER induced by thapsigargin was strongly depressed by tBHP in the absence (Figure 6b), but not the presence (Figure 6a), of extracellular calcium. Thus, tBHP has greater inhibitory effects on signaling in the absence, compared to the presence, of extracellular calcium by two measures. This might be related to changes in cellular calcium distribution associated with the stress caused by the acute removal of calcium from the extracellular medium. Luminal calcium was also depressed by exposure to tBHP for 90 min (in the presence of extracellular calcium). Apparently this decrease in [Ca^2+^]_L_ was not sufficient to reduce the initial IP3 response in the presence of extracellular calcium. It is likely that oxidative stress and the perturbation of calcium homeostasis via the removal of extracellular calcium have an additive effect on ER calcium release in response to IP3 receptor activation. This would explain the different effects of oxidative stress observed in the presence and absence of extracellular calcium.

SOCE was visualized using experimental paradigms involving depletion of calcium from the ER by either muscarinic activation of IP3 receptors or thapsigargin inhibition of ER calcium pumps in the absence of extracellular calcium. After [Ca^2+^]_i._ returned to a baseline level, calcium was reintroduced to the extracellular medium, and calcium immediately entered the cell via the SOCE channels that had been opened in response to ER calcium depletion. In both paradigms, a robust inhibitory effect of tBHP exposure on SOCE was apparent.

In contrast, no evidence was obtained for an effect of oxidative stress on proximal muscarinic receptor signaling events. Specifically, acute exposure to tBHP did not affect antagonist binding affinity, agonist binding affinity, the sensitivity of agonist binding to guanine nucleotides, or the ability of allosteric ligands to affect receptor binding kinetics. This was somewhat unexpected in light of several reports of redox and sulfhydryl reagent sensitivity of muscarinic binding properties in neuronal tissues (e.g., [29,30].

Exposure to metal oxide nanoparticles creates oxidative stress in a variety of cells, and one impetus for the present study was the inhibitory effects of metal oxide nanoparticles on signaling mediated by CHO-M3 cells [16]. ZnO nanoparticles and tBHP had several similar effects on CHO-M3 signaling [16]. Notably, ZnO and tBHP both increased [Ca^2+^]_i_ and depressed SOCE. However, ZnO had greater inhibitory effects on IP3 receptor- and thapsigargin-mediated calcium release from the ER. Moreover, ZnO had pronounced effects on agonist binding to the M3 receptor (increasing agonist affinity and eliminating guanine nucleotide sensitivity), produced a greater increase in resting [Ca^2+^]_i_, and had a greater effect on SOCE (completely eliminating SOCE at certain nontoxic concentrations). It seems likely that the effects of ZnO do not reflect a simple increase in oxidative stress produced by an hydroxyl radical generating agent.

## Conclusions

Stress caused by a potent oxidant affected several aspects of M3 receptor signaling pathway in CHO cells, including resting [Ca^2+^]_i_, [Ca^2+^]_L_, IP3 receptor mediated release of calcium from the ER, and calcium entry through the SOCE. In contrast, tBHP had little effect on M3 receptor binding or G protein coupling. Thus, oxidative stress affects multiple aspects of calcium homeostasis and calcium dependent signaling.

## Abbreviations

BSS: Basal salt solution; [Ca2+]i: Intracellular calcium concentration; [Ca2+]L: Calcium concentration in the lumen of the endoplasmic reticulum; CHO: Chinese hamster ovary; ER: Endoplasmic reticulum; IP3: Inositol trisphosphate; DCFDA: 2′,7′-dichlorodihydrofluorescein; Gpp(NH0)p: 5′-guanylyl-imidodiphosphate; [3H]MS: [^3^H] *N*-methylscopolamine; MTS: 3-(4,5-dimethylthiazol-2-yl)-5-(3-carboxymethoxyphenyl)-2-(4-sulfophenyl)-2H-tetrazolium; PLCβ: Phospholipase Cβ; SOCE: Store operated calcium entry; tBHP: *tert*-butyl hydroperoxide.

## Competing interests

The authors declare that they have no competing interests.

## Authors’ contributions

T-HT performed the ligand binding measurements and performed data analysis and reviewed the manuscript H-JW measured calcium responses and cell viability and revised the manuscript JDE, performed data analysis and reviewed the manuscript RAR and measured ER calcium concentration, C-TC, AGM, EKS and ALM measured calcium responses, performed data analysis and reviewed the manuscript Y-WH was involved in study design and preparing the manuscript RSA designed the experiments, analyzed the data and prepared the manuscript. All authors read and approved the final manuscript.
